# Super resolution live imaging: The key for unveiling the true dynamics of membrane traffic around the Golgi apparatus in plant cells

**DOI:** 10.3389/fpls.2022.1100757

**Published:** 2022-12-22

**Authors:** Yoko Ito, Tomohiro Uemura

**Affiliations:** ^1^ Institute for Human Life Science, Ochanomizu University, Tokyo, Japan; ^2^ Graduate School of Humanities and Sciences, Ochanomizu University, Tokyo, Japan

**Keywords:** super-resolution microscopy, live imaging, SCLIM, membrane traffic, Golgi apparatus

## Abstract

In contrast to the relatively static image of the plants, the world inside each cell is surprisingly dynamic. Membrane-bounded organelles move actively on the cytoskeletons and exchange materials by vesicles, tubules, or direct contact between each other. In order to understand what is happening during those events, it is essential to visualize the working components *in vivo*. After the breakthrough made by the application of fluorescent proteins, the development of light microscopy enabled many discoveries in cell biology, including those about the membrane traffic in plant cells. Especially, super-resolution microscopy, which is becoming more and more accessible, is now one of the most powerful techniques. However, although the spatial resolution has improved a lot, there are still some difficulties in terms of the temporal resolution, which is also a crucial parameter for the visualization of the living nature of the intracellular structures. In this review, we will introduce the super resolution microscopy developed especially for live-cell imaging with high temporal resolution, and show some examples that were made by this tool in plant membrane research.

## Introduction

The advances in cell biology have been propelled by breakthroughs in cellular microscopy imaging. After the first biological studies by microscopy in the 17^th^ century, which led to the discovery of the “cell”, one of the huge steps was the development and application of the electron microscopy. As we can see from the historic works from the group of George Palade, Albert Claude, and Keith Porter, the discoveries made by the electron microscopy literally formed the foundation of modern cell biology (Claude, 1975; Sabatini, 1999; Tartakoff, 2002; Satir, 2005). Since then, the electron microscopy has been contributing a lot for the investigation of membrane traffic within the cells including plant cells (Otegui and Pennington, 2019; Liu et al., 2020; Weiner et al., 2021).

However, the nature of membrane traffic is amazingly dynamic, and many of the single-membrane bounded organelles are not stable. For example, the Golgi structure is maintained in the balance of membrane and protein exchange between neighboring compartments at the early secretory pathway. Therefore, the inhibition of transport between the endoplasmic reticulum (ER) and the Golgi apparatus quickly affects the Golgi structure within 5 minutes and leads to the complete disappearance of the Golgi apparatus in almost 30 minutes in both animal and plant cells (Klausner et al., 1992; Ritzenthaler et al., 2002; Schoberer et al., 2010; Ito et al., 2012; Ito et al., 2014). Also in the endocytic pathway, the extracellularly added lipophilic dye FM4-64 becomes visible at the early endosome within only a few minutes, indicating that the small endosomal vesicles quickly carry the dye from the plasma membrane (Dettmer et al., 2006; Viotti et al., 2010). In addition, although the plants look immobile to our eyes, the intracellular components move even much faster than those in animal cells. The speed of the actomyosin dependent movement, called cytoplasmic streaming, reaches up to several micrometer per second while the size of the organelles such as Golgi or multivesicular bodies is less than 1 micrometer (Shimmen and Yokota, 2004; Nebenführ and Dixit, 2018). The plant organelles efficiently exchange materials with each other in the mid of this stream. When we want to reveal what is really going on during those trafficking processes, Achilles heel of the electron microscopy is that it requires sample fixation. This means that we can capture only one snapshot of a dynamic process. If we get an electron microscopic image of two connected membrane structures, there is no information whether they are under fusion or fission.

The biggest turning point after the electron microscopy would be the application of fluorescent proteins to the subcellular observations. The genetically encoded fluorescent tags enable the protein-specific labeling under the light microscope, meaning that we can follow the behaviors of those proteins and the organelles where they localize in living cells. This innovation explosively expanded the world of cell biology. In plant membrane trafficking research field, starting from introducing ER-localizing green fluorescent protein (GFP), fluorescent protein labeling was quickly applied not only to mark the organelles but also to visualize cargos in order to analyze the trafficking activity (Oparka et al., 1995; Boevink et al., 1996; Batoko et al., 2000).

Fluorescent live imaging is available thanks to the visible light, but the limitation of live imaging by optical microscopy also comes from the physical nature of the light. As Ernst Abbe and Lord Rayleigh formulated in the 19^th^ century, the resolution of microscopy is limited to approximately half of the wavelength of the light, meaning that objects closer than about 200 nm cannot be resolved even with perfect lenses as long as we use the visible light. In this respect, the light microscopy is far less advantageous than the electron microscopy, which uses electrons with a 10^5^ times smaller wavelength (Schermelleh et al., 2010; Prakash et al., 2022).

However, of course the biologists’ desire is in between the limits of those two microscopy technologies; observation of smaller structures in living cells. Recent efforts in the light microscopy by many approaches are actually breaking the limit. The optical microscopy techniques that overcome the diffraction limit are generally called super-resolution microscopy. Among them, the most famous are the Nobel prize-winning methods called stimulated emission depletion (STED), structured illumination microscopy (SIM), and photoactivated localization microscopy or stochastic optical reconstruction microscopy (PALM/STORM). Each of them realizes the super-resolution less than 100 nm in XY plane by unique techniques taking advantage of the physical nature of the light or fluorescent proteins (Prakash et al., 2022). In addition, microscopy companies have developed easy-to-use “soft super-resolution” methods that can be added to conventional confocal systems, such as ZEISS Airyscan with the resolution 1.7 times better than the diffraction limit (Huff, 2015). These methods are quickly evolving and becoming more and more accessible, and are already applied in many plant studies (Komis et al., 2015; Schubert, 2017; Liu et al., 2020; McGinness et al., 2022).

Nevertheless, those super-resolution methods still have some points that do not fit well with the live-cell imaging of membrane trafficking. Because usually the spatial and temporal resolutions are in a trade-off relationship, most of the super-resolution systems have some trouble in high-speed acquisition, and this is not only problematic for the observation of rapidly moving objects but also often results in photobleaching or phototoxicity. The low acquisition rate is also a bottleneck for 3D observation, while the organelles and carriers move around in 3D. Some of the systems have also a limitation in the multicolor acquisition with more than two colors, which means that for example there is a high hurdle for the observation of a cargo transported between two different organelles (Colin et al., 2021; Prakash et al., 2022).

With a demand of a super-resolution system with a more weight on the high-speed multicolor acquisition, a system named Super Resolution Confocal Live Imaging Microscopy (SCLIM) was developed (Tojima et al., 2023; Nakano, 2002; Kurokawa et al., 2013; Kurokawa and Nakano, 2020). Here, we introduce the system and its application to the actual biological studies especially in plant cells.

## SCLIM for membrane traffic research

**Figure 1:**
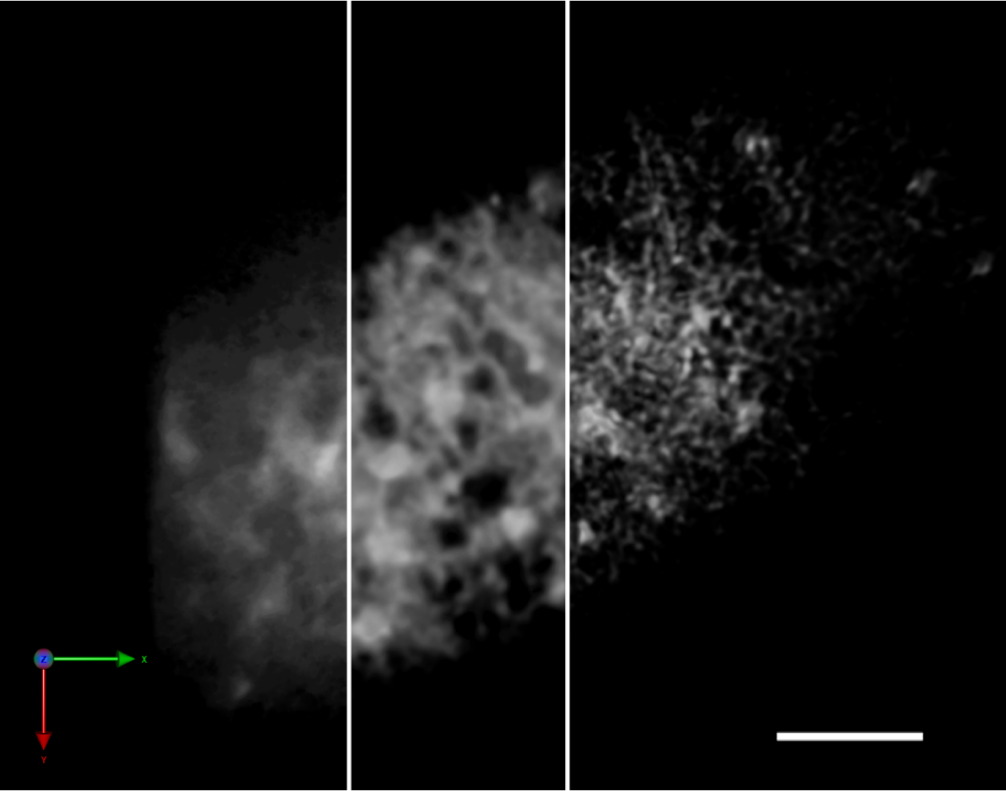
Improvement of the spatial resolution of a SCLIM image by repetitive deconvolution. 3D image of the ER in a tobacco BY-2 cell (SP-iRFP-HDEL) taken by SCLIM. The resolution was improved by the iterative restoration (repetitive deconvolution) of Volocity software. Raw image (left), after 5 iterations (middle), and after 10 iterations (right). Sacle bar = 5 μm.

SCLIM is based on the spinning-disk confocal system, the strength of which is the capacity of high-speed scanning. The bottleneck of spinning-disk scanning often comes from the low signal intensity that requires longer exposure time for the detection, which consequently reduces its advantage in speed. SCLIM is equipped with image intensifiers with a custom-made cooling system for each EM-CCD camera that achieve thousands-fold signal amplification in high signal-to-noise (S/N) ratio at video rate (30 frames/s) (Kurokawa et al., 2013). This high-sensitivity detection is the most important feature of the system. The collected images with precise information give high accuracy to the post-acquisition mathematical processing (deconvolution), which realizes the resolution 180 nm in XY that is beyond the diffraction limit of light (**Figure 1**). Additionally, acquiring 3D images with the slice-to-slice interval much smaller than the optical slice thickness can be regarded as “oversampling” that also contributes to the precise deconvolution processing. The specially designed dichroic mirror/band-pass filter set is able to separate up to five fluorescent proteins, and the cameras for each fluorescence window enable the exact simultaneous multicolor acquisition. So far in living plant cells, three color 4D (XYZ plus time) observation of GFP, RFP (red fluorescent protein), and iRFP (infra-red fluorescent protein) has been successfully performed with multiple combinations of labeled proteins (Ito et al., 2018; Shimizu et al., 2021). In short, although its spatial resolution is not extremely high as other common super-resolution methods, SCLIM is designed to show its best performance in the observations of dynamic processes with multiple players in living cells.

The first biological question that SCLIM challenged was the big controversy about the intra-Golgi trafficking. The Golgi apparatus consists of multiple membrane sacs called cisternae which usually pile up to form stacks. Stacks are clearly polarized between the *cis* and the *trans* side, and the cargos delivered from the ER travel through the organelle from *cis* to *trans*. About how the cargos are transported in the Golgi, there were two major models: one was that the cargos are packed in the vesicles that emerge from the stable cisternae and travel forward to the next cisternae (“vesicular transport”), and the other was that the cargos stay in the cisternae and the resident proteins of each cisterna are transported back to the pervious cisterna by retrograde vesicles (called “cisternal maturation” because the nature of the cisternae changes during this process) (Glick and Nakano, 2009; Nakano and Luini, 2010). Taking advantage of the yeast *Saccharomyces cerevisiae* that has the unstacked Golgi, SCLIM detected that the localizing proteins are displaced by the proteins of later cisternae within one cisterna in 4D, which was one of the first direct evidence of cisternal maturation (Losev et al., 2006; Matsuura-Tokita et al., 2006). Afterwards, the following improvement of SCLIM has made it possible to observe the cisternal maturation with the cargo stays in the same cisternae (Kurokawa et al., 2019).

With the success in yeast, SCLIM came to be applied for the membrane traffic research in other cell types including plants. The microscopy system itself is universal and can be used for various organelles (Ebine et al., 2008; Ishikawa et al., 2018), but it has been utilized the most for the observations around the Golgi apparatus. Its major contribution to the research field would be the characterization of organelle “sub-compartments” or “sub-domains/zones” around the Golgi. The compartments and some specific zones within them that were undistinguishable from the neighbors by the electron microscopy or conventional light microscopy were demonstrated to have distinct nature and dynamics.

## The dynamics of Golgi entry: GECCO as the universal intermediate at the ER-Golgi interface

At the “entry” side of the Golgi that receives materials from the ER, the electron microscopic studies have suggested that the first *cis* cisternae are formed by the collection of 3 to 5 ER-originated COPII vesicles, and those one or two newly formed *cis*-cisternae are the cargo sorting station without glycosylation activities (Donohoe et al., 2013). Our study using the ER-Golgi traffic inhibitor Brefeldin A (BFA) on tobacco BY-2 cells has demonstrated that the cisternae on the most *cis* side of the Golgi stacks behave differently upon BFA treatment; the proteins of those cisternae localize to punctate structures while the other Golgi components are absorbed into the ER. The *cis*-Golgi SNARE SYP31 and the membrane protein retrieval receptor RER1B have been identified to localize to such *cis*-most cisternae, which is reasonable from their function in the traffic at the ER-Golgi interface (Ito et al., 2012). Those cisternae are located more on the *cis* side than the ones containing α-1,2-mannosidase I (ManI), the first Golgi-α-mannosidase acting in the *N*-glycosylation chain, indicating that they would be equivalent to the sorting-specialized *cis* cisternae described in Donohoe et al. (2013). A recent study also revealed that the ER-α-mannosidase MNS3, the enzyme acts one step before ManI and important for the recognition of the misfolded glycoproteins for degradation, actually localizes not to the ER but to the Golgi and colocalizes with SYP31 at the punctate structures upon BFA treatment (Schoberer et al., 2019). SCLIM observation showed that the punctate structures after BFA treatment are not the ER exit sites (ERES) but the compartments closely associated with them (Ito et al., 2012). Moreover, 4D analysis revealed that the *trans*-Golgi protein that has relocalized to the ER is transported through the punctate structures during Golgi regeneration after BFA removal. When coexisting at the same structure, the *cis*-most- and the *trans*-Golgi proteins are not evenly mixed, similar to the SCLIM super-resolution images of maturing cisternae in yeast (Matsuura-Tokita et al., 2006; Ishii et al., 2016; Ito et al., 2018). From these results, based on the function of receiving the other Golgi components and being the scaffold for stack regeneration, we have named those punctate structures (and in the broad sense also the original *cis*-most Golgi cisternae) Golgi entry core compartment (GECCO) (Ito et al., 2018; Ito and Boutté, 2020; Nakano, 2022).

In vertebrate cells that have a radial microtubule pattern, the Golgi apparatus is concentrated close to the centrosome/microtubule organizing center. Consequently, about a half of the ERES are far apart from the Golgi and the long-distance transport is required at the ER-Golgi interface (Stephens, 2003). This is achieved by the ER-Golgi intermediate compartment (ERGIC), which is vesicular-tubular structures obviously separated from the main body of the Golgi. ERGIC-53, the protein frequently used as the ERGIC marker, is known to localize to punctate structures upon BFA treatment in mammalian cells (Lippincott-Schwartz et al., 1990). Considering the position at the entry side of the Golgi and the behavior upon BFA treatment, GECCO seems to be the plant counterpart of ERGIC, although it is not physically separated from the other cisternae. The characterization of GECCO as the Golgi sub-compartment that has a distinct nature brought us the idea that the existence of such a specialized entry compartment at the ER-Golgi boundary might be a shared feature of the eukaryotes. Indeed, also in *S. cerevisiae*, the SCLIM 4D observation revealed that only the *cis*-cisternae of the characteristic unstacked Golgi show specific “hug-and-kiss” action to receive cargoes from the ERES (described later). Further analysis of plant GECCO might contribute to unveil the universal mechanism of ER-Golgi trafficking.

## The dynamics of Golgi exit: Sorting by specialized sub-organelle zones

At the “exit” side of the Golgi, there is another vesicular-tubular compartment called *trans*-Golgi network (TGN), which produces different kinds of transport vesicles and sorts the cargos into them. TGN used to be thought as a part of the Golgi and it was often referred to just as the *trans*-Golgi. However, the localization analysis of TGN Qa-SNAREs by live imaging in *Arabidopsis* protoplasts found that TGN is sometimes separated from the Golgi (Uemura et al., 2004). Subsequent electron microscopic studies reported that some TGN-like structures seem to be detaching from the main Golgi stack, and proposed that the late TGN cisternae become independent organelle as they mature and get fragmented into vesicles in the end of their life (Staehelin and Kang, 2008; Kang et al., 2011). Finally, live-cell imaging by spinning disc confocal microscopy and also 4D super-resolution imaging by SCLIM revealed that the plant TGN shows apparently independent movement from the *trans*-Golgi and not only dissociate but also associate with the Golgi reversibly, indicating that the existence of “the Golgi-independent TGN (GI-TGN)” reflects more than the one-way maturation (Viotti et al., 2010; Uemura and Nakano, 2013; Uemura et al., 2014). Together with the fact that the plant TGN functions also as the early endosome (Dettmer et al., 2006; Lam et al., 2007; Chow et al., 2008; Viotti et al., 2010), the striking data of the TGN dynamics made the idea of the compartment as an independent organelle widely accepted in the plant research field.

In addition to this change of our view of the TGN, super-resolution live imaging has contributed to the characterization of TGN sub-domains or functional zones. Since the TGN is the intracellular trafficking hub where multiple transport routs intersect, a complex cargo sorting is taking place within this compartment. The idea that the plant TGN is not homogeneous and can be divided into some sub-domains that produce specific carriers for efficient cargo sorting emerged around the start of this millennium. Beginning with an immunoelectron microscopic research (Bassham et al., 2000), a number of studies have reported that some trafficking-related TGN proteins including coat proteins, SNAREs, and tethering factors show segregated localizations (Chow et al., 2008; Gendre et al., 2011; Boutté et al., 2013; Wattelet-Boyer et al., 2016; Ravikumar et al., 2018; Heinze et al., 2020), and the electron tomography revealed that there are at least two types of vesicles budding from the TGN (Kang et al., 2011). The SCLIM observation also added some distinctly localizing TGN proteins, and moreover, taking advantage of the simultaneous triple-color super-resolution system, they were clearly shown to reside in different sub-regions within one TGN labeled by another protein (Shimizu et al., 2021). This revealed that there are at least two zones in the TGN, one with the proteins mediating secretory traffic and the other with those mediating vacuolar traffic. SCLIM also displayed its ability in the analysis of the dynamics of those TGN zones. Portions of the secretory-trafficking zone were observed to leave the TGN while this did not happen with the vacuolar-trafficking zone, suggesting that the GI-TGN is involved in the secretory trafficking pathway (Shimizu et al., 2021; Shimizu and Uemura, 2022).

## Microscopic approaches for the remaining problems in plant early secretory pathway

Albeit the development of the super-resolution microscopy including SCLIM, there are still some biological questions that cannot be covered either by the electron or by the light microscopy. For example, one of the membrane traffic problems of the early secretory pathway in plants that is left in this technical valley is that whether there are COPII vesicles or not. As it is widely known, the COPII coat and its regulatory mechanism is very well conserved among the eukaryotes and working at the ER-to-Golgi anterograde traffic, but whether those vesicles budded from the ERES are really pinched off and become free is still under debate. The controversy comes from the fact that the number of electron microscopic studies that captured the images of COPII vesicles in higher plants is very limited (Hawes, 2012; Robinson et al., 2015; Robinson, 2020). Even the COPII budding profiles are difficult to be observed in higher plants and have been reported only by high-pressure frozen samples (Ritzenthaler et al., 2002; Donohoe et al., 2006; Kang and Staehelin, 2008; Staehelin and Kang, 2008), while the classical chemical fixation sufficiently works for algae such as *Chlamydomonas* (Hummel et al., 2007). This have been tried to be explained by multiple possibilities; the COPII buds might be highly unstable and the vesicles could be also very rapidly consumed (Staehelin and Kang, 2008), or ER-Golgi transport might be achieved not mainly by vesicles but by direct tubular connections as it has been suggested in mammalian cells for the transport of large cargos such as procollagen (Raote and Malhotra, 2019). Another possibility is that the Golgi cisternae temporarily approach to the COPII budding sites and directly capture the cargos from them before the vesicles float away from the ER. SCLIM observations of the dynamics of the Golgi cisternae and the COPII coat plus cargo transport in yeast cells have demonstrated that the *cis*-cisternae come close toward the ERES, remain associated for a few seconds and then move away, thus this movement was given the name “hug-and-kiss”. During this “kissing” period, the intensity of the COPII coat at the contact area often decreases and the cargo is loaded to the *cis*-Golgi, indicating the COPII uncoating and the membrane connection for cargo transfer (Kurokawa et al., 2014). For the fluorescence live imaging in plant cells, COPII coat proteins are often used to label the ERES, but actually it is not currently possible to tell whether the structures labeled by COPII coat are the budding sites on the ER or the clusters of coated free vesicles. Because the size of the COPII vesicles is typically about 60 nm and would be mobile, any of the current light microcopy do not have sufficient spatiotemporal resolution yet. Also, as most of the COPII buds are shown to be within only 300 nm from the Golgi in *Arabidopsis* cells, it is still difficult to see the dynamic change of ER-Golgi relation like hug-and-kiss if it is taking place in plants as well (Kang and Staehelin, 2008; Staehelin and Kang, 2008).

Another problem of current organelle live-imaging based on fluorescent proteins is that what we can see is just the proteins and not the membranous structures themselves. As it is apparent from the findings about TGN zones, one kind of protein does not necessarily label the whole structure uniformly even if the membrane is connected. This is also the case for the ER-Golgi interface. Although it was believed that Sar1, the COPII coat assembly regulator, presented all over the COPII buds to keep the coat on the membrane before, recent studies in yeast cells or *in vitro* reconstitution assay using yeast proteins showed that Sar1 localizes only to the rim of the budding region (Kurokawa et al., 2016; Iwasaki et al., 2017). This indicates the possibility of partial uncoating during the COPII budding. Also in mammalian cells, it is suggested that the COPII coat remains only at the neck of the ERES and functions as the gatekeeper for cargo sorting into ERES, therefore the region labeled by COPII coat might not be the ERES *per se* (Shomron et al., 2021). One solution would be the correlative light and electron microscopy (CLEM), the combination of electron and fluorescent microscopies. A recent study of HeLa cells using focused ion beam scanning electron microscopy (FIB-SEM) combined with super-resolution cryogenic-SIM has shown the 3D ultrastructure of ERES with the distribution of COPI, COPII, and cargo proteins, which was totally different from the classical image of ERES (Hoffman et al., 2020; Weigel et al., 2021). CLEM is beginning to be applied to the endomembrane structures in plant cells as well, and is under improvement thanks to the development of related methods such as recovering of more fluorescent signal after fixation or automatic system to support correlation (Toyooka and Shinozaki-Narikawa, 2019; Wang et al., 2019). Although there are still some difficulties in sample preparation for applying the same 3D electron microscopy methods like FIB-SEM to vascular plants because of the plant-specific reasons such as the large cell size and the presence of huge vacuoles (Liu et al., 2020; Weiner et al., 2021), technical advancement would overcome them. Nevertheless, as the dynamics information is lost in CLEM, the improvement in light microscopy is yet required. Imperfect though it is, visualization of more kinds of proteins at the same time would bring more information.

## Future perspectives

**Figure 2:**
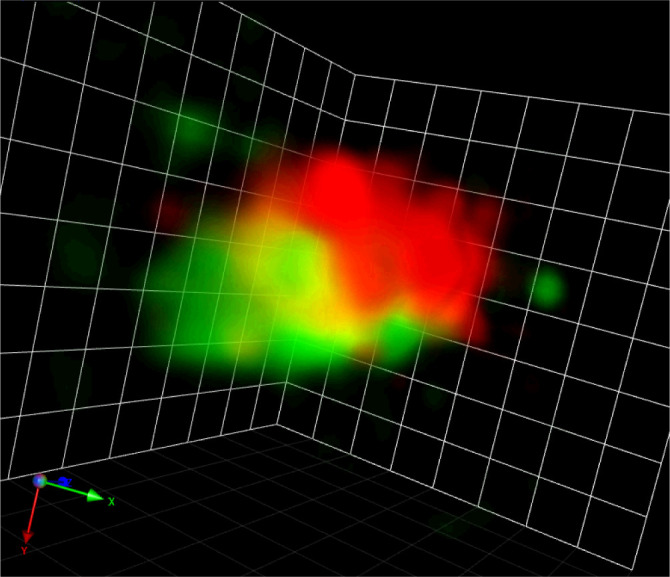
A Golgi stack visualized by SCLIM-2M. A snapshot of a 3D movie of a Golgi stack visualized by GFP-SYP31 (*cis*) and ST-mRFP (medial/*trans*) in a tobacco BY-2 cell captured by SCLIM-2M. Grid = 441 nm.

The co-evolution of microscopic techniques and cell biology continues running. Regarding SCLIM, since the first-generation system (SCLIM-1) was originally designed for the observation of small yeast cells, the field of view is sometimes too small for other cell types including plants. Therefore, the advanced version called SCLIM-2K (K for the developer Yasuhito Kosugi) with sCMOS cameras for a wider field of view has been developed and already in actual use for mammalian cells (Tojima et al., 2023). Its 150 nm XY resolution is better than SCLIM-1 and the operation software became user-friendly, so it will be a good tool for plant cell observation. In addition, SCLIM is evolving into another way as well, to achieve much higher spatiotemporal resolution. This system, called SCLIM-2M (M for the developer Daisuke Miyashiro), is equipped with ultrafast cameras (1000 frames/s) and enables single-photon localization. With the originally built deconvolution algorithm, our preliminary data of the fluorescent Golgi markers in BY-2 cells already shows many tiny vesicles less than 100 nm diameter moving around the Golgi stacks (**Figure 2**).

Not only the microscopy system but also recent progresses in fluorescent protein and the cargo transport visualizing methods are already showing their power in the animal research field (Boncompain et al., 2012; Chen et al., 2013; Hirano et al., 2022). It would be in the close future that we can open the next door of plant membrane trafficking research with the advanced imaging technology.

## Author contributions

YI wrote the manuscript and prepared the figures. TU edited the manuscript. All authors contributed to the article and approved the submitted version.
